# Methyltransferase like 3-mediated N6-methylatidin methylation inhibits vascular smooth muscle cells phenotype switching *via* promoting phosphatidylinositol 3-kinase mRNA decay

**DOI:** 10.3389/fcvm.2022.913039

**Published:** 2022-10-28

**Authors:** Yongchao Zhao, Aichao Xia, Chaofu Li, Xianping Long, Zhixun Bai, Zhimei Qiu, Weidong Xiong, Ning Gu, Youcheng Shen, Ranzun Zhao, Bei Shi

**Affiliations:** ^1^Department of Cardiology, Affiliated Hospital of Zunyi Medical University, Zunyi, China; ^2^Department of Nephrology, The Second Affiliated Hospital of Zunyi Medical University, Zunyi, China

**Keywords:** neointimal hyperplasia, VSMCs phenotype switching, N6-methyladenosine, methyltransferase like 3, mRNA decay

## Abstract

N6-methylatidine (m6A) is involved in post-transcriptional metabolism and a variety of pathological processes. However, little is known about the role of m6A in vascular proliferative diseases, particularly in vascular smooth muscle cells (VSMCs) phenotype switching-induced neointimal hyperplasia. In the current study, we discovered that methyltransferase like 3 (METTL3) is a critical candidate for catalyzing a global increase in m6A in response to carotid artery injury and various VSMCs phenotype switching. The inhibited neointimal hyperplasia was obtained after *in vivo* gene transfer to knock-down *Mettl3*. *In vitro* overexpression of *Mettl3* resulted in increased VSMC proliferation, migration, and reduced contractile gene expression with a global elevation of m6A modification. In contrast, *Mettl3* knockdown reversed this facilitated phenotypic switch in VSMCs, as demonstrated by downregulated m6A, decreased proliferation, migration, and increased expression of contractile genes. Mechanistically, *Mettl3* knock-down was found to promote higher phosphatidylinositol 3-kinase (*Pi3k*) mRNA decay thus inactivating the PI3K/AKT signal to inhibit VSMCs phenotype switching. Overall, our findings highlight the importance of METTL3-mediated m6A in VSMCs phenotype switching and offer a novel perspective on targeting METTL3 as a therapeutic option for VSMCs phenotype switching modulated pathogenesis, including atherosclerosis and restenosis.

## Introduction

Atherosclerosis, which includes coronary atherosclerosis, restenosis, and other vascular proliferative diseases, is still the leading cause of morbidity and mortality throughout the world (1, 2). The transition of vascular smooth muscle cells (VSMCs) from a differentiated phenotype (contractile cells) to a dedifferentiated phenotype (synthetic cells) has been shown to play an important role in the pathogenesis of atherosclerosis (3). To maintain vascular tone, VSMCs in normal, healthy vessels have a low proliferative and migratory rate and express a variety of contractile-related genes. VSMCs exhibit highly dedifferentiated phenotypes in response to mechanical or chemical vascular injury, characterized by robust proliferation, migration, and decreased expression of contractile-related genes, such as alpha-smooth muscle actin (α*-Sma*), smooth muscle protein 22-alpha (*Sm22*α), etc., which eventually lead to neointimal hyperplasia (4). The underlying mechanism that controls this phenotype switching, however, is not yet fully understood.

The epigenetic dimensional modulation of gene expression is centrally involved in regulating VSMCs pathogenesis, according to evidence from numerous studies (5). Abnormalities in DNA methylation (6, 7), histone modification (8), and non-coding RNAs (9, 10) have been studied extensively among the identified epigenetic mechanisms in this process, whereas, studies on post-transcriptional chemical modifiers of RNA have received little attention. N6-methylatidine (m6A) is the most common type of eukaryotic RNA modification among the more than 150 discovered to date (11). The m6A represents a dynamic and reversible process that is activated by methyltransferase 3 (METTL3), methyltransferase 14 (METTL14), methyltransferase 16 (METTL16), and Wilms tumor 1-associated protein (WTAP) defined as “writers,” inactivated by demethylases fat mass and obesity-associated (FTO) and alk homolog 5 (ALKBH5) defined as “erasers,” recognized by YTH domain families (YTHDF1-3) and insulin-like growth factor binding proteins (IGFBP1-3) defined as “readers” (12). While, much is known about how m6A affects various biological functions of post-transcriptional RNA metabolism, such as RNA splicing (13, 14), nuclear processing (15), stability (16), decay (17), translation (18), and the development and progression of various diseases (19–21).

However, currently very little is known about the biological function and related molecular mechanism of m6A in VSMCs phenotype switching. The current study investigated the role of METTL3-mediated m6A in VSMCs phenotype switching and the post-transcriptional regulatory mechanism that controls it. METTL3, the core of methyltransferase, was screened out and found to be responsible for the global increase of m6A based on several VSMCs phenotype switching models *in vivo*, *in vitro*, and *ex vivo*. *Mettl3* knockdown caused a decrease in m6A, phenotype switching in synthetic VSMCs, and neointimal hyperplasia. Mechanistically, the decreased stability and increased decay of m6A on phosphatidylinositol 3-kinase (*Pi3k*) mRNA causes the protein kinase B (AKT) pathway to be inactivated, preventing VSMCs phenotype switching. Our research, taken together, sheds new light on the role of post-transcriptional modification in the modulation of vascular proliferative diseases, including atherosclerosis and restenosis.

## Materials and methods

All animal experiments were conducted following the National Academy of Sciences’ “Guide for the Care and Use of Laboratory Animals,” which was approved by the Animal Care and Utilization Committee at Zunyi Medical University. The Supplementary material contains a detailed description of the primers, antibodies, and small interfering RNAs (siRNAs) used in this study.

### Balloon-injured carotid arteries

The balloon injuries were performed in rat carotid arteries using male 8-week-old Sprague-Dawley rats weighing 220–260 g (Hunan SJA Laboratory Animal Co., Ltd.) as previously described (22). The left common carotid arteries were fully exposed after the rats were anesthetized. At the distal end of the external carotid artery, the common and internal carotid arteries were clamped and ligated. At the proximal end of the ligation site, an incision of about 0.25 cm in diameter was made. The common carotid artery was inserted into this incision while the vascular clamp on the common carotid artery was removed. To successfully damage the vascular endothelium, a 2.0 mm × 20 mm balloon (Medtronic, Inc.) was inflated to 3.5 atmospheres and rotated five times counterclockwise and five times clockwise. The balloon was then deflated, and the incision was closed. The injured arteries were harvested 14 days after surgery for further testing.

### Hematoxylin-eosin staining and immunofluorescence

The arteries were paraffin-embedded and cut into 5 μm slices. The intimal and medial areas were measured and calculated after the sections were stained with hematoxylin-eosin (HE) staining solution and visualized under a microscope. Deparaffinized sections were deparaffinized and blocked for 1 h with five percent goat serum (Beyotime, #C0265) for immunofluorescence. The primary antibodies made with goat serum were dropped and refrigerated overnight at 4°C. The secondary antibodies were then added dropwise and incubated for 1 h at room temperature, protected from light. After that, the images were visualized in fluorescence microscopy using the DAPI staining solution for another 10 min. Supplementary Table 1 provides a detailed description of the antibodies used in this study.

### RNA extraction and real-time quantitative polymerase chain reaction

Total RNA was extracted by a columnar RNA extraction kit (Sangon, #B511321-0100) according to the manufacturer’s instructions. After that, 1,000 ng of total RNA was reverse transcribed (Takara, #RR036A). The total system was 20 μl, including 4 μl of 5 × PrimeScript RT Master Mix, 10 μl of 100 ng/μl total RNA, and 6 μl of DEPC solution (Bioshap, #BL510A). Amplification was conducted on a CFX Connect Real-Time System (Bio-Rad, CA, USA) using an amplification kit (Takara, #RR820A). The total system was 10 μl, including 5 μl TB Green Premix Ex Taq II, 0.5 μl forward primers, 0.5 μl reverse primers, 0.8 μl template DNA, and 3.2 μl DEPC. RT-qPCR reaction program settings have been composed of: 95°C for 30 s, 95°C for 5 s, and 60°C for 30 s for 39 cycles. After normalizing the expression of the gene to β-actin, the relative expression of the target gene was calculated using the 2^–Δ^ ^Δ^ *^CT^* method. Primer sequences are shown in Supplementary Table 2.

### Dot blot

The DEPC (Bioshap, #BL510A) solution was used to adjust total RNA concentrations to 50 ng/μl and 100 ng/μl, respectively. Total RNA was serially diluted and denatured at 95°C for 3 min before being cooled on ice. Two microliters total RNA was carefully dropped onto a Hyond-N^+^ membrane (GE Healthcare, #RPN203B), dried at room temperature, and then cross-linked to the Hyond-N^+^ membrane using the UV cross-linking instrument (1.2 J × 3 min). The membrane was blocked for 1 h at room temperature in a 5% BSA (Epizyme, #PS133) before being incubated overnight at 4°C in a specific anti-m6A antibody dilution buffer (Supplementary Table 1). After that, the secondary goat anti-rabbit antibody (Proteintech, #SA00001-2) was incubated for another 1 h. In a Bio-Rad X-ray developer system, the results were obtained. The membrane was then stained for 5 h at room temperature with 0.1% methylene blue (MB). Images were visualized after washing the membrane with ddH_2_O to see if total RNA loading was consistent. Image J software was used to perform a quantitative analysis of the bands’ gray values.

### Western blot

The proteins were extracted and separated using the Epizyme (#PG112) kit before being transferred to a PVDF membrane (Epizyme, #WJ001). For 10 min, a protein-free rapid-blocking solution (Epizyme, #PS108) was used to block the cells. The primary antibody was incubated at 4°C overnight before being rinsed three times for 10 min with Tris-buffered saline containing Tween 20 (TBST). The secondary antibodies were then incubated at room temperature for another 1 h. The Bio-Rad X-ray development system was used to obtain the results after the ECL luminescent substrate (Tanon, #180-501) was added dropwise to the membrane (Bio-Rad, CA, USA). The loading control was -Actin, and the data were analyzed using Image J software. The antibodies used for Western Blot in this study are listed in Supplementary Table 1.

### Vascular smooth muscle cells isolation and platelet-derived growth factor treatment

Vascular smooth muscle cells were cultured as described previously (23). In brief, cervical dislocation was performed on SD rats aged 6 weeks. The aortic explants were removed and soaked in a four percent penicillin/streptomycin solution (Solarbio, #P1400) twice. The adventitia was then dissected by the cuff after the perivascular tissue was removed. The medial tissue was cut longitudinally, the intima was gently scraped off, and the cells were then placed in DMEM supplemented with 20% FBS (MRC, #CCS300090). The obtained vascular medial tissue was minced into 1 mm^3^ piece and transferred to a culture flask containing 3 ml DMEM supplemented with 20% FBS, which was then inverted for 2 h before being flipped. On day 3, cells crawled out around the tissue block, and the solution was changed on day 5. The blocks were washed off and the cells were passaged once they had reached 85% confluence. For the following experiments, passages 3–5 were chosen. For 48 h, cells were treated with 20 ng/ml platelet-derived growth factor (PDGF-BB) (R&D, #520-BB) to induce phenotype switching in VSMCs. To assess PDGF-BB-induced VSMCs phenotype switching, the expression of proliferation, migration and contractile genes were examined.

### 5-Ethynyl-2’-deoxyuridine, scratch, and transwell assay

The proliferation of VSMCs was detected using the EdU kit (Beyotime, #C0071S). Briefly, the EdU solution was diluted in a 1,000:1 culture medium before being added to 24-well plates seeded with 300 μl of cells per well. Put it back in the incubator for another 2 h. Fixative (Biosharp, #BL539A) was used to fix the cells, and 0.4% TritonX-10 (Biosharp, #BS084) was used to permeabilize. The reaction solution was added at 500 μl per well after rinsing twice with PBS. Incubate in the dark for another 30 min. A fluorescence microscope was used to photograph and record a 100 μl per well DAPI (Solarbio, #C0065) solution incubated for 5 min. A scratch assay was utilized to assess VSMCs migration. In a 6-well plate, the VSMCs were grown to full confluence before being wounded with a sterile 200 μl pipette tip. 2 ml of DMEM culture medium containing 0.1% FBS was added after rinsing twice with PBS. The plates were then returned to the incubator for another 48 h. Under an inverted microscope, images were captured and the scratch area of VSMCs was calculated using Image J software. A transwell assay was further conducted to assess VSMCs migration. Briefly, a 24-well Boyden chamber with a porous polycarbonate membrane (8 μm pore size; Corning, NY, USA) was used by us to examine cell migration function. The cells treated with different conditions were seeded in the upper chamber (100 μl/well, 5 × 10^4^ cells/ml), and the lower chamber was filled with DMEM culture media containing 10% FBS (500 μl/well). After incubation for 12 h (5% CO_2_, 37°C), the cells that migrated through the filter membrane were fixed with 4% paraformaldehyde for 15 min and stained with crystal violet (#070920201103) for 30 min. Then the number of migrated cells was photographed and recorded under a microscope.

### Delivery of adeno-associated virus

The detailed carotid artery gene delivery method was performed as previously described (24). In brief, an adeno-associated virus (AAV) (Hanbio Co., Ltd.) containing *Sm22a*, a specific VSMCs promoter, was created to knock-down Mettl3 expression on VSMCs with greater specificity. Then, in a liquid state, 1 × 10^12^ v.g./ml control AAV (*AAV-shCtrl*) or AAV to knock-down *Mettl3* (*AAV-shMettl3*) were mixed into 30 percent pluronic F-127 (PF-127) glue (Sigma, #9003-11-6). The carotid arteries of 4-week-old male SD rats weighing 60–80 g were then fully exposed, and PF-127 glue was evenly wrapped around the common carotid artery. The balloon injury was performed 4 weeks later, and samples were taken for further investigation on day 14 after surgery.

### siRNA transfection

Vascular smooth muscle cells were seeded into 6-well plates to reach a confluence of 50% before transfection. Then, 5 μl of 20 μM *Mettl3* siRNA (*siMettl3*) (Hanbio, #PK210802121DS) or negative control siRNA (*siNC)* (#JX211009) was diluted into 200 μl in serum-free medium, and mix well with the RNAFit (HANBIO, #HB-RF-500) at room temperature for 10 min. Subsequently, 1.8 ml of culture medium containing 10% FBS with 200 μl mixture per well were placed into a 6-well plate. The silencing efficiency was tested by RT-qPCR and Western Blot at 24 h after transfection. The sequence of siRNAs used in this study is shown in Supplementary Table 3.

### Methylated RNA immunoprecipitation-qPCR

The MeRIP-qPCR was carried out according to Millipore’s instructions (#17-10499). VSMCs were lysed with RIP buffer and immunoprecipitated in IP buffer overnight with specific anti-m6A antibody or control IgG antibody-conjugated beads, followed by RNA purification. Finally, RT-qPCR was used to determine the enrichment of m6A immunoprecipitated RNA, which was normalized to the input control. Supplementary Table 4 lists the primers used in this study for MeRIP-qPCR.

### Detection of mRNA half-life

Vascular smooth muscle cells were seeded in 6-well plates and each well was treated with a 5 μl stock solution (1 mg/ml) of actinomycin D (Sigma, # 50-76-0). Following that, total RNA was extracted at time points of 0, 1, 2, 4, 6, and 8 h after adding actinomyces D, with steps referring to total RNA extraction. Following that, the RT-qPCR was carried out, and all of the groups were normalized using a Ct value reference of 0 h. The relative mRNA decay rate and half-life were calculated using GraphPad Prism 9.0 software and non-linear regression curve fitting (single-phase decay).

### Statistical analysis

The data were presented as a mean standard ± deviation (SD). When the data satisfies a normal distribution, the Shapiro-Wilk test was used to determine normality, and the differences between the two groups were compared using two-tailed unpaired student’s *t*-tests; otherwise, the Mann-Whitney non-parametric test was used. For multiple groups, a one-way ANOVA with Bonferroni’s *post-hoc* test was used to determine statistical significance. GraphPad Prism 9 (San Diego, CA, USA) was used for statistical analysis, and a *P*-value of less than 0.05 was considered significant.

## Results

### Methyltransferase like 3 is up-regulated in vascular smooth muscle cells phenotypic switch *in vivo, in vitro, ex vivo* and is involved in global N6-methylatidine upregulation

A rat carotid artery balloon injury model was used to induce neointimal hyperplasia to explore if m6A was involved in VSMCs phenotype switching. The intima-to-media area ratio of harvested carotid increased significantly 14 days after balloon injury, according to morphology analysis of HE staining (Supplementary Figure 1A). Further immunofluorescence indicated reduced levels of α-SMA and SM22α (contractile marker proteins) and greater levels of OPN (synthetic marker protein) and PCNA (proliferative marker protein) (Supplementary Figures 1B,C). These findings suggested that a successful neointimal hyperplasia model was created.

**FIGURE 1:**
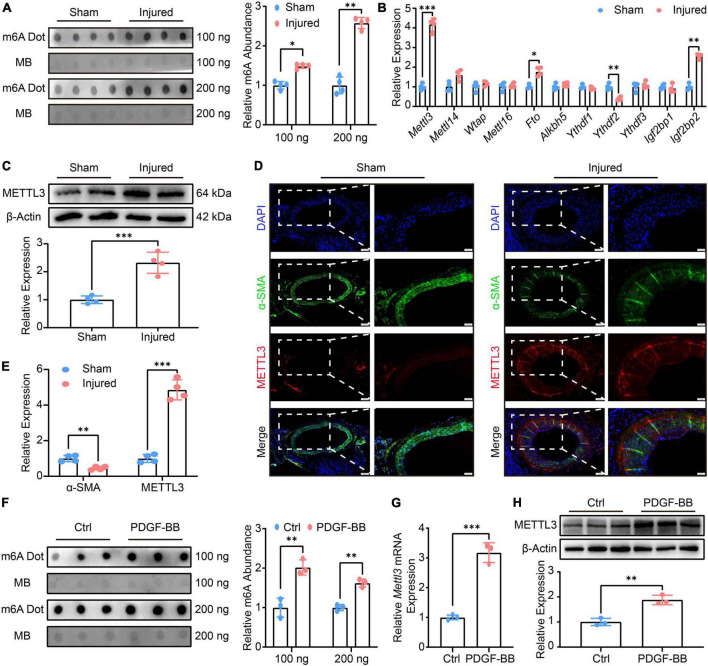
Methyltransferase like3 (METTL3) is up-regulated in phenotype switched vascular smooth muscle cells (VSMCs) *in vivo, in vitro*, and *ex vivo* and is involved in global N6-methylatidine (m6A) upregulation. **(A)** Representative m6A Dot Blot image (left panel) and quantification (right panel) of sham-operated and balloon-injured carotid arteries 14 days after surgery (*n* = 4). **(B)** Quantitative analysis of m6A-related genes detected by RT-qPCR (*n* = 4). **(C)** Representative Western blot images (upper panel) and quantification (lower panel) of METTL3 (*n* = 4). **(D,E)** Representative immunofluorescence images and quantification showing METTL3 and α-SMA expression (scale bar = 100 μm). **(F)** Representative Dot Blot image (left panel) and quantification (right panel) showing VSMCs m6A modification treated with or without PDGF-BB (*n* = 3). **(G)** RT-qPCR quantification of *Mettl3* expression (*n* = 3). **(H)** Representative Western Blot image (upper panel) and quantitative analysis (lower panel) of METTL3 level. Data are presented as mean ± SD. *p < 0.05, ^**^p < 0.01 and ^***^p < 0.001.

Total RNAs were extracted after balloon injury, and m6A was detected using a Dot Blot assay. The findings revealed a global rise in m6A modification (Figure 1A). The expression of m6A-related methyltransferases, demethylases, and recognition proteins was comprehensively examined using RT-qPCR to investigate the factors that led to the upward revision of m6A (Figure 1B). *Mettl3*, *Fto*, *Ythdf2*, and *Igf2bp2* were found to be differentially expressed in the RT-qPCR analysis. Among these genes, *Mettl3*, as a protein level further collaborated by Western Blot (Figure 1C), was the most aggressively up-regulated. Considering that METT3 is a methyltransferase and the m6A was found to be up-regulated globally, despite the fact that the expression of the demethylase *Fto* was also significantly increased. METTL3 was finally chosen as a candidate for further investigations. The location of METTL3 in neointimal hyperplasia was then investigated further (Figure 1D). When METTTL3 was overlaid with α-SMA, another synthetic VSMCs manufacturer, the immunofluorescence image revealed that METTTL3 was highly expressed in the neointima-to-media area (Figure 1E), implying that METTL3 could be a key regulator in VSMCs phenotype switching.

Given that PDGF-BB is a well-known platelet-derived bioactive mediator that causes VSMCs to switch from a contractile cell phenotype to a highly synthetic and proliferating cell type that promotes injury repair (25). Thus, the VSMCs were cultured and indentified by immunofluorescence (Supplementary Figure 1D). Next, the proliferation and migration of VSMCs treated by PDGF-BB were also assessed. The EdU assay revealed a higher number of EdU positive VSMCs in PDGF-BB-induced conditions (Supplementary Figure 1E), implying that PDGF-BB-induced VSMCs proliferation. The scratch assay and transwell assay were both utilized to test the VSMCs migratory ability, and the results showed a higher scratch closure and migration in PDGF-BB-treated VSMCs (Supplementary Figures 1F,G), indicating that PDGF-BB-induced VSMCs migration. Finally, the expression of VSMCs marker genes was investigated, and RT-qPCR data revealed a decreased expression of α*-Sma*, *Cnn1*, and *Sm22*α, as well as an increased expression of *Opn*, *Myh10, and Vim*, in the PDGF-BB stimulation, compared to the Ctrl (Supplementary Figure 1H). After that, we examined the m6A modification and METTL3 expression *in vitro* VSMCs phenotype switching induced by PDGF-BB. The m6A was found to be significantly up-regulated in VSMCs after PDGF-BB treatment (Figure 1F), and both *Mettl3* expression (Figure 1G) and METTL3 level (Figure 1H) were significantly increased. In conclusion, PDGF-BB-treated VSMCs resulted in an increase in m6A and a promoted switch from contractile to the synthetic phenotype.

To further comprehensively elucidate the conserved nature of high *Mettl3* expression pattern and enhanced m6A level in VSMCs phenotypic switching. Three phenotypic switching models were constructed and confirmed by RT-qPCR: 20% fetal bovine serum (FBS)-induced rat VSMCs *in vitro* (Supplementary Figure 2A), rat VSMCs with 3rd to 9th passages of extended culture *in vitro* (Supplementary Figure 2D), and cultured rat thoracic aortas *ex vivo* (Supplementary Figure 2G). In either 20% FBS-induced VSMCs (Supplementary Figures 2B,C), continuous passaged VSMCs (Supplementary Figures 2E,F), or cultured aortas (Supplementary Figures 2H,I), these additional findings collectively demonstrated a global increase in m6A and a higher expression of *Mettl3*. Overall, these findings revealed a conserved signature *in vivo*, *in vitro*, and *ex vivo* for increased m6A modification and METTL3 level, implying a critical regulatory role of METTL3 for VSMCs phenotype switching.

### Reduced N6-methylatidine and neointimal hyperplasia are associated with methyltransferase like 3 downregulation

**FIGURE 2:**
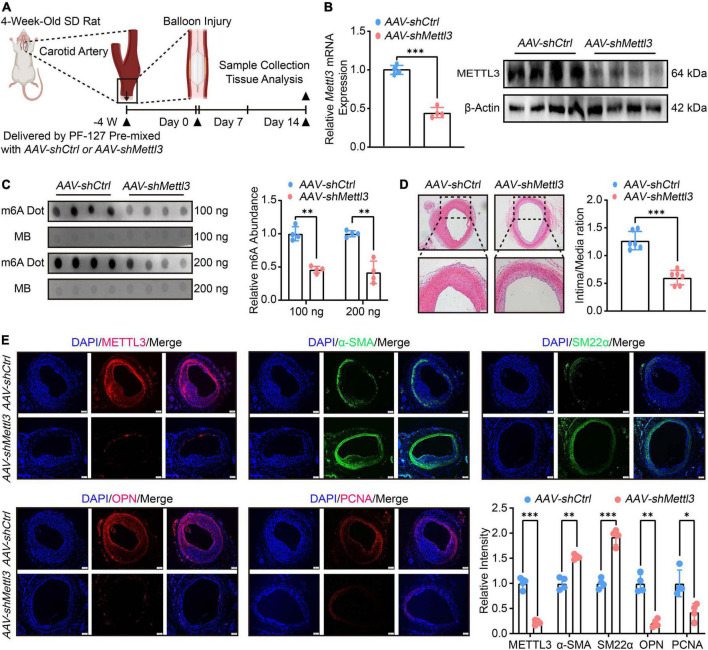
Reduced N6-methylatidine (m6A) and neointimal hyperplasia are associated with methyltransferase like 3 (METTL3) downregulation. **(A)** Schematic illustration of the experimental procedure. The carotid arteries of 4-week-old SD rats were treated with PF-127 mixed with *AAV-shCtrl* or *AAV-shMettl3*. Four weeks later, the rats were subject to balloon injured. 14 days after surgery, samples were harvested for further investigations. **(B)** Quantification analysis of *Mettl3* expression measured by RT-qPCR and Western Blot images showing METTL3 level after delivery of AAV and subject to carotid artery balloon injury. **(C)** Dot Blot image (left panel) and relative m6A abundance quantification (right panel). **(D)** Representative HE staining images (left panel) and quantification (right panel) of intima area (*n* = 6, scale bar = 100 μm). **(E)** Representative immunofluorescence images showing METTL3, α-SMA, PCNA, OPN, and SM22α levels (scale bar = 100 μm). Three randomly selected fields for each sample were visualized. Data were from at least four independent biological replicates unless specified and presented as mean ± SD. *p < 0.05, ^**^p < 0.01 and ^***^p < 0.001.

To assess the role of METTL3-mediated m6A in neointimal hyperplasia, an adeno-associated virus (AAV) containing *Sm22a*, a specific VSMCs promoter, was constructed to knock-down *Mettl3* expression. The carotid arteries of 4-week-old rats were treated with *AAV-shCtrl* and *AAV-shMettl3* delivered by PF-127. On day 14 after the injury, the balloon injury was operated on and the carotid arteries were harvested for further analysis (Figure 2A). The RT-qPCR and Western Blot results confirmed that both *Mettl3* expression and METTL3 levels were significantly down-regulated (Figure 2B and Supplementary Figure 3A). To explore whether m6A was altered in the presence of METTL3 downregulation. Total RNAs of carotid were extracted and the Dot Blot assay revealed a significant reduction in global m6A when compared to the control (Figure 2C).

To determine whether there is a link between low METTL3 levels and neointimal hyperplasia. The intima thickness of *AAV-shMettl3* treated carotid arteries was significantly inhibited, according to HE staining (Figure 2D). The immunofluorescence results showed that reduced METTL3 expression was linked to thin intima thickness, increased contractile markers level of α-SMA and SM22α and decreased synthetic markers level of OPN and PCNA (Figure 2E). In line with this phenomenon, the RT-qPCR data also found a significant increase in contractile gene expression, including α*-Sma*, *Cnn1* and *Sm22*α, and a significant decrease in synthetic gene expression, including *Opn*, *Myh10*, and *Vim* (Supplementary Figure 3B). Overall, the evidence strongly suggests that METTL3 deficiency reduced m6A modification and neointimal hyperplasia.

### Methyltransferase like 3 promotes vascular smooth muscle cells phenotype switching

To explore the role of METTL3 in VSMCs *in vitro*, the effects of METTL3 gain-of-function and loss-of-function on VSMCs phenotype switching induced by PDGF-BB were investigated. Firstly, the VSMCs were transfected with either control lentivirus (*Lv-Ctrl*) or lentivirus specific to *Mettl3* (*Lv-Mettl3*). The RT-qPCR (Supplementary Figure 4A) and Western Blot (Supplementary Figures 4B,C) analysis confirmed a stable up-regulation of METTL3 level, which was associated with a significant global increase in m6A (Supplementary Figures 4D,E) in either vehicle or PDGF-BB-treated cells. The proliferation and migration of VSMCs were also assessed. The EdU assay revealed that up-regulated METTL3 was associated with a higher number of EdU positive VSMCs only in PDGF-BB-induced conditions, not in vehicle-induced conditions (Supplementary Figure 4F), implying that *Mettl3* overexpression promoted PDGF-BB-induced VSMCs proliferation. The scratch assay and transwell assay were used to test the migratory ability, and the results showed that upregulated *Mettl3* expression resulted in a higher scratch closure and migration cells in PDGF-BB-treated VSMCs, indicating that increased METTL3 significantly augment PDGF-BB-induced migration. Interestingly, this phenomenon was not observed in vehicle-treated VSMCs even though *Mettl3* expression was up-regulated (Supplementary Figures 4G,H). Finally, the expression of VSMCs-related genes was investigated, and RT-qPCR data revealed a decrease in α*-Sma*, *Cnn1* and *Sm22*α, as well as an increase in *Opn*, *Myh10*, and *Vim*, in the *Lv-Mettl3* group in response to PDGF-BB stimulation, compared to *Lv-Ctrl* group (Supplementary Figure 4I). In conclusion, METTL3 overexpression resulted in an increase in m6A and a further promoted phenotype switch of VSMCs.

**FIGURE 3:**
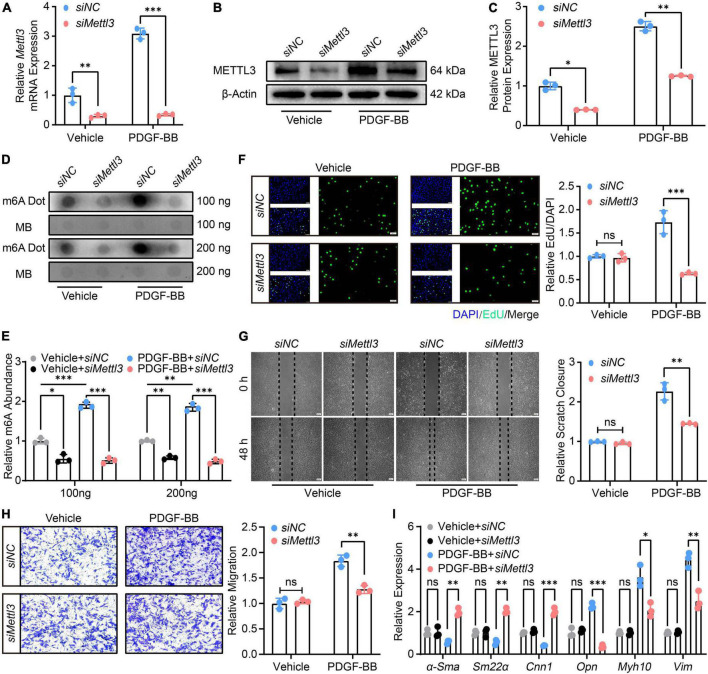
Down-regulation of methyltransferase like 3 (METTL3) results in decreased N6-methylatidine (m6A) and prevents vascular smooth muscle cells (VSMCs) from phenotype switching. **(A)** Quantitative analysis of *Mettl3* expression transfected with control siRNA (*siNC*) and siRNA target to *Mettl3* (*siMettl3*) upon PDGF-BB present or not. **(B,C)** Representative Western Blot image **(B)** and quantification data **(C)** showing METTL3 expression. **(D,E)** Representative Dot Blot image **(D)** and quantitative analysis **(E)** of m6A level. **(F)** Representative EdU images [(left panel), scale bar = 50 μm] and quantification of VSMCs proliferation (right panel). Three randomly selected fields for each sample were acquired. **(G)** Representative scratch images [(left panel), scale bar = 200 μm] and quantification of VSMCs migration (right panel). Three randomly selected fields for each sample were acquired. **(H)** Representative transwell images [(left panel), scale bar = 100 μm] and quantification of VSMCs migration (right panel). Three randomly selected fields for each sample were acquired. **(I)** Quantitative RT-qPCR analysis of contractile genes α*-Sma*, *Sm22*α, *Cnn1*, and synthetic genes *Opn*, *Myh10*, and *Vim* expression. All data were from three independent biological replicates and presented as mean ± SD. *p < 0.05, ^**^p < 0.01 and ^***^p < 0.001 and ns indicates no significance.

The VSMCs were then transfected with either control siRNA (*siNC*) or siRNA specific to *Mettl3* (*siMettl3*). The RT-qPCR (Figure 3A) and Western Blot (Figures 3B,C) analysis confirmed a stable down-regulation of METTL3 level, which was associated with a significant global decrease in m6A (Figures 3D,E) in either vehicle or PDGF-BB-treated cells. The proliferation and migration of VSMCs were also assessed. The EdU assay revealed that down-regulated METTL3 was associated with a lower number of EdU positive VSMCs only in PDGF-BB-induced conditions, not in vehicle-induced conditions (Figure 3F), implying that *Mettl3* knockdown inhibited PDGF-BB-induced VSMCs proliferation. The scratch assay and transwell assay was used to test the migratory ability, and the results showed that knocking down *Mettl3* resulted in a lower scratch closure and migration cells in PDGF-BB-treated VSMCs, indicating that decreased METTL3 significantly reduced PDGF-BB-induced migration. Interestingly, this phenomenon was not observed in vehicle-treated VSMCs even though *Mettl3* expression was down-regulated (Figures 3G,H). Finally, the expression of VSMCs-related genes was investigated, and RT-qPCR data revealed an increase in α*-Sma*, *Cnn1*, and *Sm22*α, as well as a decrease in *Opn*, *Myh10, and Vim*, in the *siMettl3* group in response to PDGF-BB stimulation, compared to vehicle-treated VSMCs (Figure 3I). In conclusion, METTL3 deficiency resulted in a decrease in m6A and a promoted switch reversal from synthetic to contractile phenotype in VSMCs.

### Methyltransferase like 3-mediated N6-methylatidine deficiency promotes phosphatidylinositol 3-kinase mRNA decay thus inactivating PI3K/AKT signaling

**FIGURE 4:**
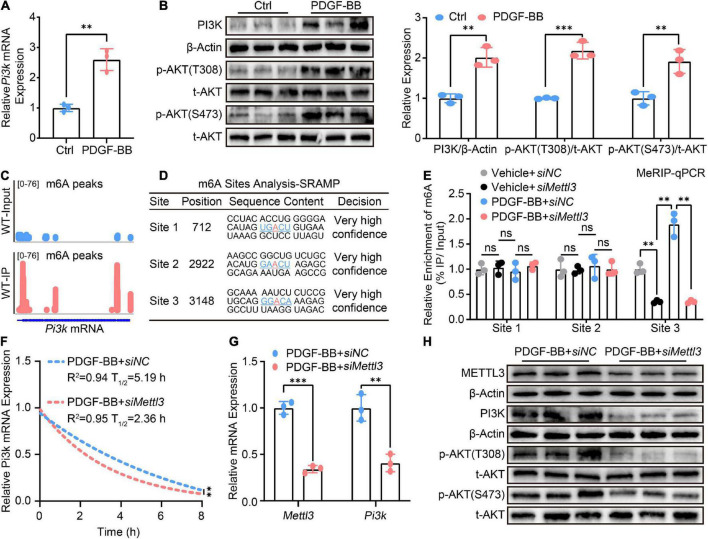
Methyltransferase like 3 (METTL3)-mediated N6-methylatidine (m6A) deficiency promotes *Pi3k* mRNA decay thus inactivating PI3K/AKT signaling. **(A)** RT-qPCR quantification of *Pi3k* expression before and after treatment of VSMCs with PDGF-BB. **(B)** Representative Western Blot image and quantitative analysis of PI3K p-AKT(T308) and p-AKT(S473) levels. **(C)** Integrated genome browser (IGV) display of m6A peak distribute on *Pi3k* mRNA from the GES94148 dataset. **(D)** The m6A modification sites prediction results of *Pi3k* mRNA from the online SRAMP database. **(E)** MeRIP-qPCR quantitative analysis m6A fold enrichment of *Pi3k* mRNA detected by primers designed specifically for three sites predicted upon the transfection of *siNC* or *siMettl3*. **(F)** Non-linear regression analysis of RT-qPCR showing the half-life of *Pi3k* mRNA. **(G)** Quantitative analysis of *Pi3k* mRNA expression. **(H)** Western Blot detection image of METTL3, PI3K, p-AKT(T308), and p-AKT(S473) proteins. All data were from three independent biological replicates and presented as mean ± SD. *p < 0.05, ^**^p < 0.01, and ^***^p < 0.001 and ns indicates no significance.

Until now, the mechanical evidence for the effect of METTL3 on VSMCs phenotype switching is still lacking. Numerous previous studies have shown that the PI3K/AKT pathway plays a critical role in VSMCs phenotype switching (26–28). RT-qPCR was used to confirm the exact role of the PI3K/AKT signal in VSMCs switching, and it revealed that *Pi3k* expression was significantly up-regulated in PDGF-BB-stimulated VSMCs phenotype switching (Figure 4A). AKT phosphorylation at Thr308 (p-AKTT308) and Ser473 (p-AKTS473) were both increased (Figure 4B), indicating that PI3K/AKT signaling was indeed activated upon PDGF-BB-induced phenotype switching. However, it was unclear whether the PI3K/AKT signal is regulated by METTL3-mediated m6A.

To test the hypothesis, we used the gene expression omnibus (GEO) database to seek the presence of m6A on *Pi3k* mRNA. The methylated RNA immunoprecipitation with next-generation sequencing (MeRIP-seq) data from the GES94148 dataset revealed that m6A modifications do distribute on *Pi3k* mRNA, as shown by the tool of integrated genome browser (IGV) (Figure 4C). As previously reported, although m6A mRNA methylation preferably occurs within the RRACH consensus motif, only a small portion of such RRACH sites are modified (29, 30). Based on the identified mammalian m6A sites in single-nucleotide resolution from the sequence-based RNA adenosine methylation site predictor (SRAMP) online database^1^ (31). Three m6A sites with a high level of confidence were screened for the possible presence of *Pi3k* mRNA (Figure 4D). Three pairs of primers specific to different m6A sites were designed and the methylated RNA immunoprecipitation qPCR (MeRIP-qPCR) assay was established to confirm the effect of METTL3 deletion on m6A modification of *Pi3k* mRNA. In METTL3 deficient VSMCs, only a decrease in m6A modification at site 3 within *Pi3k* mRNA was observed (Figure 4E).

Because the presence of m6A methylation impacts the post-transcriptional RNA metabolism including RNA stability and decay (16). Actinomycin D, a transcriptional inhibitor cultured with VSMCs for a different time point, was used to test the decay rate of *Pi3k* mRNA. The RT-qPCR assay revealed that lower METTL3 was associated with a shorter half-life of *Pi3k* mRNA, implying that the loss of METTL3-mediated m6A deletion of *Pi3k* mRNA preferred more rapid decay (Figure 4F). This crucial mechanistic evidence explained the characteristic, down-regulated *Pi3k* mRNA expression detected by RT-qPCR (Figure 4G) and PI3K protein level detected by Western Blot (Figure 4H), resulting in PI3K/AKT pathway inactivity as evidenced by decreased phosphorylation of p-AKT(T308) and p-AKT(S473).

### Methyltransferase like 3 regulates the phenotype switching of vascular smooth muscle cells is phosphatidylinositol 3-kinase-dependent

**FIGURE 5:**
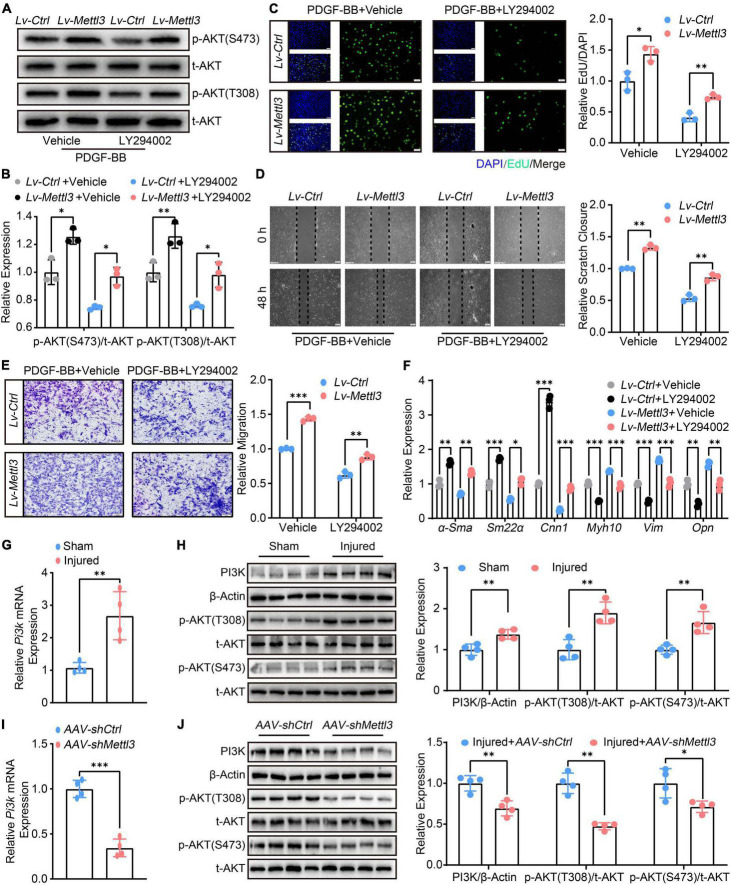
Methyltransferase like 3 (METTL3) regulates the phenotype switching of vascular smooth muscle cells (VSMCs) is PI3K-dependent. **(A,B)** Representative Western Blot images showing the impact of LY294002 on p-AKT(T308) and p-AKT(S473) expression upon *Lv-Ctrl* or *Lv-Mettl3* transfection. **(C)** Representative EdU images [(left panel), scale bar = 50 μm] and quantitative analysis (right panel) of LY294002 treating or not on VSMCs proliferation. Three randomly selected fields for each sample were acquired (*n* = 3). **(D)** Representative Scratch images [(left panel), scale bar = 200 μm] and quantitative analysis (right panel) of LY294002 treating or not on VSMCs migration. Three randomly selected fields for each sample were acquired (*n* = 3). **(E)** Representative transwell images [(left panel), scale bar = 100 μm] and quantification of VSMCs migration (right panel). Three randomly selected fields for each sample were acquired. **(F)** Quantitative RT-qPCR analysis of α-*Sma*, *Sm22*α, *Cnn1*, *Myh10*, *Vim*, and *Opn* expression (*n* = 3). **(G)** Quantitative analysis of *Pi3k* mRNA expression in carotid arteries subjected to balloon-injured or sham-operated (*n* = 4). **(H)** Western Blot image (left panel) and quantification (right panel) of PI3K, t-AKT, p-AKT(T308), and p-AKT(S473) levels from the carotid arteries subject to balloon-injured and sham-operated (*n* = 4). **(I)** Quantitative analysis of *Pi3k* mRNA expression in carotid arteries subjected to balloon-injured or sham-operated after *AAV-shCtrl* or *AAV-shMettl3* delivery (*n* = 4). **(J)** Western Blot image (left panel) and quantification (right panel) of PI3K, t-AKT, p-AKT(T308), and p-AKT(S473) proteins from the carotid arteries subject to balloon-injured and sham-operated after *AAV-shCtrl* or *AAV-shMettl3* delivery (*n* = 4). Data are presented as mean ± SD. *p < 0.05, ^**^p < 0.01, and ^***^p < 0.001.

Although the deficiency of Mettl3 led to the post-transcriptional decay of *Pi3k* mRNA, it was unclear whether METTL3-modulated VSMCs phenotype switching was dependent on PI3K. LY294002, a specific PI3K inhibitor, was used to restrain PI3K phosphorylation. Following that, the effect of METTL3-induced phosphorylation on AKT was investigated. The results showed that LY294002 blocked METTL3 overexpression-induced activation of p-AKT(T308) and p-AKT(S473), implying that LY294002 countered PI3K/AKT activation by up-regulating METTL3 (Figures 5A,B). The effects of LY294002 on METTL3-mediated VSMCs proliferation, migration, and relative contractile gene expression were also investigated. The EdU assay demonstrated a significant reduction in the number of proliferative VSMCs (Figure 5C), as well as a reduction in the scratch closure area and the number of migratory VSMCs (Figures 5D,E). Furthermore, the RT-qPCR analysis revealed a significant increase in α*-Sma*, *Sm22*α, *Cnn1*, and a significant decrease in *Opn*, *Myh10*, *Vim* (Figure 5F). Overall, these findings showed that restraining PI3K phosphorylation could block the function of the PI3K/AKT signal and VSMCs phenotype switching, implying that METTL3 modulated VSMCs function is PI3K dependent.

To support the necessity of *in vivo* carotid artery balloon injury, we also examined the expression of *Pi3k* and phosphorylation of PI3K/AKT signal. The RT-qPCR (Figure 5G) and Western Blot (Figure 5H) confirmed that *Pi3k* expression was up-regulated and the PI3K/AKT signal was activated upon balloon injury. In the injured carotid artery with AAV-mediated loss of METTL3, the results found that *Pi3k* was declined (Figure 5I) and p-AKT(T308), p-AKT(S473) were decreased (Figure 5J). Overall, these findings suggest that the PI3K/AKT signal may play an equal role in METTL3-mediated m6A modulating neointimal hyperplasia *in vivo*, which warrants more investigation.

## Discussion

Prior studies have emphasized the importance of VSMCs phenotype switching in modulating neointimal hyperplasia thus protecting the initiation and progression of vascular proliferative diseases, including atherosclerosis and restenosis (3). The m6A methylation serves as an important dimensionality of epigenetic regulation of gene expression networks (32), but its role and related mechanism in VSMCs phenotype switching-induced intimal hyperplasia remain to be illustrated. In the current study, we discovered that the m6A methyltransferase METTL3 plays a critical role in regulating VSMCs phenotype switching and neointimal hyperplasia *in vivo*, *in vitro*, and *ex vivo*. The current findings show that METTL3 is a key m6A candidate involved in VSMCs phenotype switching in a variety of settings, including *in vivo* carotid artery injury, *in vitro* PDGF-BB, FBS, and passage aged stimulated VSMCs, and *ex vivo* isolated cultured thoracic aortas, and that it is responsible for the global increase in m6A modification. In terms of function, *Mettl3* deficiency protects against *in vivo* carotid artery injury and *in vitro* PDGF-BB-induced VSMCs phenotype switching, as well as a global reduction in m6A methylation. Inversely, *Mettl3* overexpression aggravates PDGF-BB-induced VSMCs phenotype switching *in vitro*, and a global augment likewise in m6A methylation. *Pi3k* mRNA was shown to be the key downstream substrate regulated by METTL3-mediated loss of m6A and has a shorter half-life post-transcriptionally to the inactive PI3K/AKT pathway, thus mitigating VSMCs phenotype switching induced by PDGF-BB. We also investigated the PI3K/AKT signal changes and discovered that decreased METTL3 mediated by AAV is linked to a lower level of PI3K and AKT phosphorylation. To the best of our knowledge, this is the first study to show the connection between METTL3-mediated m6A and VSMCs phenotype switching.

Vascular endothelial cell dysfunction serves as another important cell fraction in response to atherosclerosis (33). Jian et al. (34) found a high level of METTL14 expression in tumor necrosis factor-alpha-induced endothelial cells, indicating that METTL14 may have therapeutic potential in the treatment of endothelial dysfunction. In the rat carotid artery injury model, the current study suggests that METTL14 expression is not significantly altered, but METTL3 expression is significantly up-regulated. Furthermore, as previously mentioned, METTL3 expression is up-regulated in various *in vitro* and *ex vivo* VSMCs phenotype switching experiments. In this study, we discovered a global increase in m6A following carotid artery balloon injury. METTL3 was shown to be a significantly up-regulated component among the m6A related-methyltransferases when compared to the other methyltransferases. It is worth mentioning that after carotid artery injury, the m6A demethylase FTO was upregulated, and recognition proteins of YTHDF2 and IGF2BP2 were also differentially expressed. These findings show that m6A-related methyltransferases have variable expression patterns in various tissues or cells. Given the favorable connection in our experimental setting between the most significantly elevated METTL3 level and the m6A mutation. As a result, METTL3 was ultimately screened out as a candidate for further inquiry, while the functions of FTO, YTHDF2, and IGF2BP2 in neointimal hyperplasia were not investigated. This is a significant unmet need that will be further investigated in the near future. Nevertheless, among all the m6A modification-related methyltransferases discovered until recently, the METTL3 and METTL14 were reported to form N6-methyltransferase complex that methylates various RNAs m6A containing N (6) positions (35). Yet structural and functional studies subsequently confirm that METTL3 constitutes the catalytic core in the heterodimer complex formed with METTL14 (36, 37). Together, this evidence combined with our data confirms the core role of METTL3 as a catalytic m6A and provides a side note on the importance of regulating METTL3 in neointimal hyperplasia.

Another intriguing finding is that, despite a decrease in global m6A, we do not discover a regulated phenotype switching in non-PDGF-BB treated VSMCs *in vitro*, including proliferation, migration, and contractile marker gene expression. The specific pathological stimulus could be one explanation for this inconsistency. Phenotype switching does not occur under physiological conditions, even when m6A is altered, suggesting that m6A may regulate vascular biology homeostasis. Unlike METTL3, ALKBH5 is another m6A demethylase that can be up-regulated in hypoxic conditions, as another of our studies has shown (38). The demethylase ALKBH5 requires molecular oxygen to function, and its m6A demethylase activity is dramatically reduced in hypoxic microenvironments (39), which explains its absence under physiological conditions. As a result, it appears that METTL3-modulated VSMCs phenotype switching is specialized in certain pathological states, emphasizing the therapeutic potential of targeting METTL3 in the treatment of diseased states, such as neointimal hyperplasia.

**FIGURE 6:**
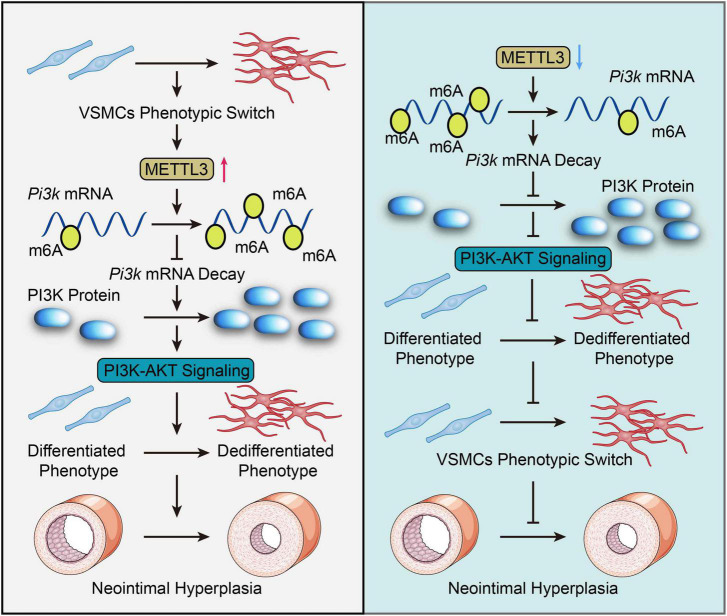
Graphic summary. In response to vascular smooth muscle cell (VSMC) phenotypic change, methyltransferase like 3 (METTL3) is dramatically up-regulated and catalyzes a global increase in m6A. METTL3 increased the m6A modification of Pi3k mRNA, which hindered its degradation and activated the PI3K-AKT pathway, thereby promoting VSMCs phenotypic switch and neointimal hyperplasia. Inversely, Mettl3 knockdown reversed this facilitated phenotypic switch in VSMCs, as demonstrated by downregulated m6A-dependent decay of Pi3k mRNA thus inactivating the PI3K/AKT signal to inhibit VSMCs phenotype switching and neointimal hyperplasia. Overall, this study highlights the importance of METTL3-mediated m6A in VSMCs phenotype switching and offers a novel perspective on targeting METTL3 as a therapeutic option for VSMCs phenotype switching modulated pathogenesis, including atherosclerosis and restenosis.

Overall, the current study still finds that METTL3 plays a critical regulatory role in neointimal hyperplasia by inhibiting VSMCs phenotype switching via post-transcriptional down-regulation of Pik3 mRNA decay in an m6A-dependent manner (Figure 6). Our findings suggest that targeting METTL3 in vascular proliferative diseases like atherosclerosis and restenosis could be a viable option. More pre-clinical and clinical studies with various VSMCs phenotype insults are needed to validate the therapeutic potential of blocking METTL3 in our settings.

### Study limitations

Although m6A methylation and METTL3 expression were investigated in other *in vitro* VSMCs phenotype switching models, the role and mechanism of METTL3-mediated m6A were only examined in PDGF-BB-induced VSMCs phenotype switching in this study. Given that different *in vitro* stimuli mimic different pathological states of phenotype switching, our findings on METTL3-modulated VSMCs induced by PDGF-BB warrant further research. In the present study, the role of METTL3 was based on the knockdown or overexpression strategies. More helpful, a convincing approach to the catalytic deficient mutant of METTL3 should be applied in the future. Moreover, PI3K is a kinase, although the gene and protein expression were changed through m6A modification, it remains unclear how m6A affected mRNAs and ultimate protein expression on the phosphorylation of PI3K. Given that the p-PI3K is regulated by the post-translational phosphorylation way and *Pi3k* mRNA is regulated by the post-transcriptional way. Future research is needed to investigate the association between these two-dimensional gene expression networks regulating pathways. Furthermore, once the m6A modification is methylated or demethylated, the biological function of post-transcriptional RNA metabolism is mediated by “reader” proteins selectively recognizing the m6A site containing the “RRACH” motif (40, 41). However, in the current study, it is unclear which “reader” is involved in the recognition of *Pi3k* mRNA, and future research is needed to clarify this issue.

## Data availability statement

The datasets presented in this study can be found in online repositories. The names of the repository/repositories and accession number(s) can be found in the article/Supplementary material.

## Ethics statement

The animal study was reviewed and approved by the Animal Care and Utilization Committee at Zunyi Medical University.

## Author contributions

BS, RZ, and YZ designed the study. YZ, AX, CL, and XL completed most of the experimental process. ZB, ZQ, and WX carried out parts of the experiments. NG and YS analyzed the data and performed the statistical analysis. BS, RZ, and YZ wrote and revised the manuscript, with contributions from AX, CL, XL, ZB, ZQ, WX, NG, and YS. All authors reviewed and approved the final manuscript.

## Footnotes

1
http://www.cuilab.cn/sramp
