# Supplementary material for: Methyltransferase like 3-mediated N6-methylatidin methylation inhibits vascular smooth muscle cells phenotype switching via promoting phosphatidylinositol 3-kinase mRNA decay
Source: Front Cardiovasc Med. 2022 Oct 28;9:913039. doi: 10.3389/fcvm.2022.913039 (PMC9649646; doi:10.3389/fcvm.2022.913039)
Supplement: Supplementary file 1 [file Data_Sheet_1.docx]

Supplementary Material

# Supplementary Materials and Methods

**VSMCs Phenotype Switching Induced by 20% FBS or Extended Culture**

By inducing VSMCs with high concentrations of serum and natural subculture, a phenotype switching model was established using VSMCs obtained through tissue attachment culture. In summary, after 24 hours of treatment with a high-glucose DMEM medium containing 20% FBS, RT-qPCR was used to detect the expression of contractile-related genes to confirm VSMC phenotype switching. In vitro, VSMCs undergo phenotype switching was also established in the natural subculture to passage 9 (P 9) and P3 and P9 of VSMCs were chosen to detect the expression of contractile-related genes to confirm phenotype switching success.

**Fresh Aortas and Cultured Aortas**

The preliminary steps for establishing the isolated culture of thoracic aortas phenotype switching were the same as for the culture of primary VSMCs from SD rats, with the exception that one part was used as a control group (Fresh Aortas) to extract RNA directly after vascular shearing, and the other part was transferred to a 6-well plate supplemented with DMEM high-glucose medium containing 20% FBS and incubated for 3 days (Cultured Aortas). To confirm the success of phenotype switching, total RNAs were extracted and the expression of contractile-related genes and synthetic-related genes in the two groups of samples were detected using RT-qPCR.

**Lentivirus Transfection**

The METTL3 overexpression in VSMCs by transfection with lentivirus (from GENE Co., Ltd.), and the experiment was performed according to the recommended protocol. In brief, 10 μl of 1 × 10^8^ TU/ml *Lv-Mettl3* (#GXDL0295097) or negative control (*Lv-Ctrl*, #LVCON335) were diluted into 980 μl of DMEM medium containing 10% FBS. After added HiTransG A (10 μl/well, GENE, #13489A3), the 6-well were placed in the incubator for continuous culture. Puromycin at 5 μg/ml was added to it 48 h after the completion of transfection, and the overexpression effect was tested by RT-qPCR and Western Blot after an additional 48 h of culture.

# Supplementary Figures and Tables

## Supplementary Tables

**S Tab. 1 Antibodies used in this study**

| Antibody specificity | Manufacture | Cat. No. | Species | Dilution |
| --- | --- | --- | --- | --- |
| Primary Antibodies for Immunofluorescence | | | | |
| Anti-α-SMA | Proteintech | 67735-1-Ig | Mouse | 1:250 |
| Anti-METTL3 | Proteintech | 15073-1-AP | Rabbit | 1:250 |
| Anti-SM22α | Proteintech | 60213-1-Ig | Mouse | 1:50 |
| Anti-OPN | Proteintech | 22952-1-AP | Rabbit | 1:50 |
| Anti-PCNA | Proteintech | 10205-2-AP | Rabbit | 1:50 |
|  | | | | |
| Secondary antibodies for Immunofluorescence | | | | |
| Anti-Rabbit IgG (CoraLite 594) | Proteintech | SA00013-4 | Goat | 1:250 |
| Anti-Mouse IgG (CoraLite 488) | Proteintech | SA00013-1 | Goat | 1:250 |
|  | | | | |
| Primary Antibody for m6A dot blot | | | | |
| Anti-m6A (Dot blot) | CST | 56593S | Rabbit | 1:1000 |
|  | | | | |
| Secondary Antibody for m6A dot blot | | | | |
| Anti-Rabbit IgG | Proteintech | SA00001-2 | Goat | 1:7000 |
|  | | | | |
| Primary antibodies for western blot | | | | |
| Anti-METTL3 | Abcam | ab195352 | Rabbit | 1:1000 |
| Anti-PI3K | Proteintech | 20584-1-AP | Rabbit | 1:500 |
| Anti-t-AKT | HUABIO | ET1609-47 | Rabbit | 1:1000 |
| Anti-p-AKT (S473) | Proteintech | 28731-1-AP | Rabbit | 1:2000 |
| Anti-p-AKT (T308) | Immunoway | YP0590 | Rabbit | 1:1000 |
| Anti-β-Actin | Proteintech | 20536-1-AP | Rabbit | 1:2500 |
|  | | | | |
| Secondary antibodies for western blot | | | | |
| Anti-Rabbit IgG | Proteintech | SA00001-2 | Goat | 1:5000 |

**S Tab. 2 Primers for RT-qPCR used in this study**

| Genes | Species | Sequence 5'-3' | |
| --- | --- | --- | --- |
| *Pi3k* | Rat | F | TGTCTTGCCTCGGAACA |
|  |  | R | CAAACTCCAGCCACACATT |
| *Mettl3* | Rat | F | GCGATGTGATTGTAGCTGAGGT |
|  |  | R | ATAACTCAATCTTGCGGGTGC |
| *β-Actin* | Rat | F | TCAAGATCATTGCTCCTCCTGAG |
|  |  | R | ACATCTGCTGGAAGGTGGACA |
| *Sm22α* | Rat | F | CTCTCCTTCCAGCCCACAAAC |
|  |  | R | ATCAGGGCCACACTGCATTA |
| *Opn* | Rat | F | TGAGGCTATCAAGGTCATCCC |
|  |  | R | TGTGTTTCCACGCTTGGTTC |
| *α-Sma* | Rat | F | TCAGCAAACAGGAGTATGACGA |
|  |  | R | TGTGCTAGAGACAGAGCGGG |
| *Mettl14* | Rat | F | GACACCCAAGTTTGATGTGATTC |
|  |  | R | CCACGTCCAGCACTTCTCGT |
| *Wtap* | Rat | F | AAGATGACCAACGAAGAACCTCTT |
|  |  | R | CCGTGCCATAACTTTGAAGTCTG |
| *Mettl16* | Rat | F | TGTTGTCTTCCCACAACCCC |
|  |  | R | CAGTGGACGGACAGATTGCT |
| *Fto* | Rat | F | CACTTGGCTTCCTTACCTGACC |
|  |  | R | GCCTCTCGGAAAACCAGTTT |
| *Alkbh5* | Rat | F | GGGTGTCGGAACCTGTGCTT |
|  |  | R | CCTGAGGCCGTATGCAGTGA |
| *Ythdf 1* | Rat | F | GGTATCCATCATTGCTGACCTG |
|  |  | R | GCACTAAACTATGGAACGCTTGTC |
| *Ythdf 2* | Rat | F | AAGGTTGAGTCTGTAAGGGTGGT |
|  |  | R | TTCTTAGGACTCCGAGCACTTTAC |
| *Ythdf3* | Rat | F | CAATGGAACAGGCAGTGAAAAC |
|  |  | R | GGATGCACCTCTACACTAGAAGGT |
| *Igfbp1* | Rat | F | AGAAATGGAAGGAGCCCTG |
|  |  | R | CATCTCCTGCTTTCTGTTGAG |
| *Igfbp2* | Rat | F | GTCCTCTGGAACATCTCTACTC |
|  |  | R | ACATCTTGCACTGTTTGAGG |
| *Myh10* | Rat | F | CAAGGTTCCCACTCCAAG |
|  |  | R | CATCAGCCACTCATCCG |
| *Vim* | Rat | F | AGGTGGAGAGGGACAACC |
|  |  | R | GGTCAAGACGTGCCAGAG |
| *Cnn1* | Rat | F | GCATGACGGTGTATGGG |
|  |  | R | TTGTGTGGGTGGTGATTG |

**S Tab. 3 List of the sequences of METTL3 siRNAs**

| Gene Symbol | Species | Sequence |
| --- | --- | --- |
| *siNC* | None | None |
| *siMettl3* | Rat | GCAGAGCAGGACUUGACUATT |

**S Tab. 4 Primers for meRIP-qPCR used in this study**

| Sites | Species | Sequence 5'-3' | |
| --- | --- | --- | --- |
| Site 1 | Rat | F | AGAAGGAGCGAGAGGAAG |
|  |  | R | AACCAGCCAATATCTTCAGG |
| Site 2 | Rat | F | GACCTGTGAACTGAGCTG |
|  |  | R | GTCATCCAACAGTCCGAG |
| Site 3 | Rat | F | TTAAAGCCACAACACACAAC |
|  |  | R | AGCGTTAACAAGCACCAT |

## Supplementary Figures and Figure Legend


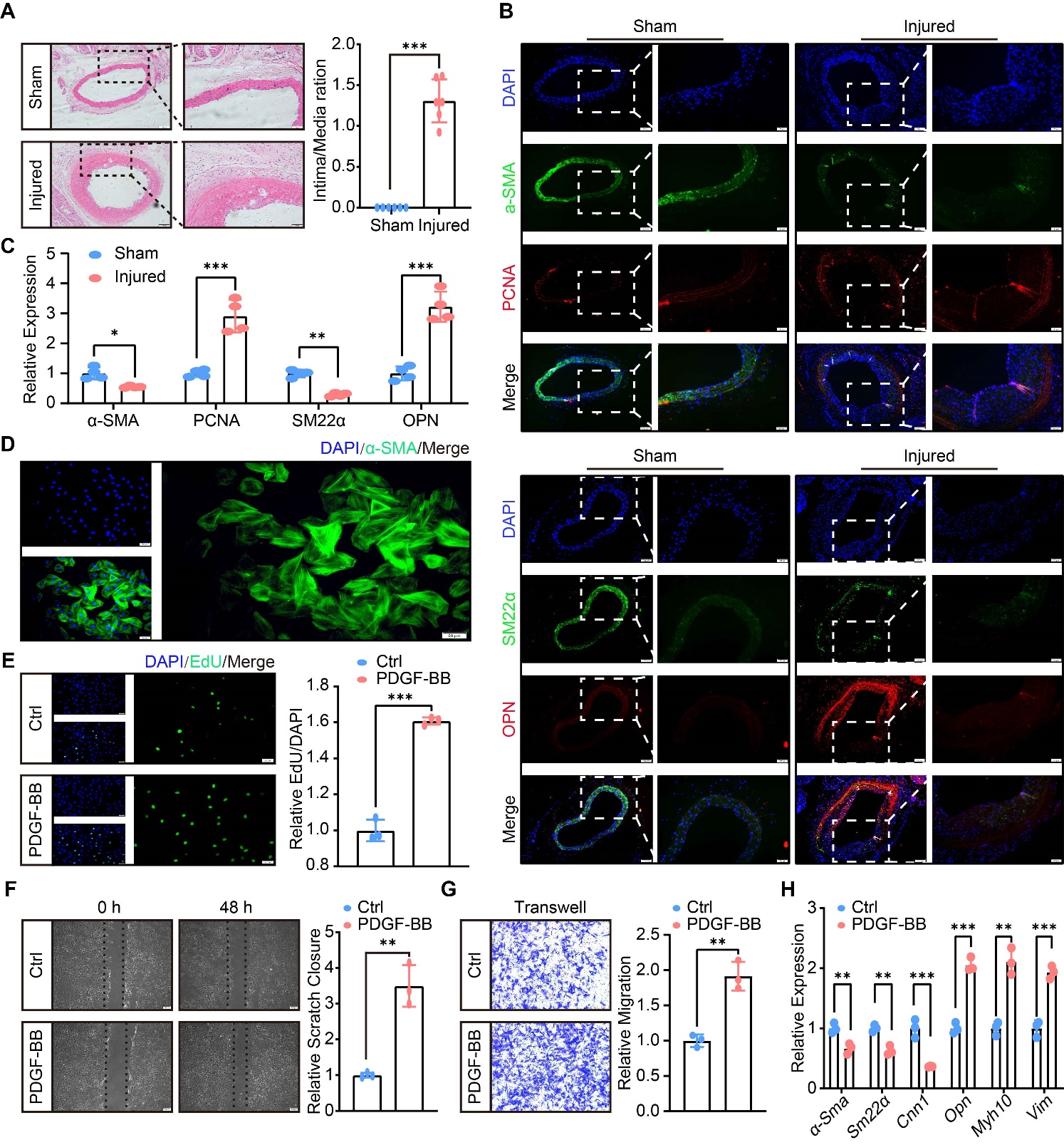


**Supplementary Figure 1.** (**A**) Representative HE staining images (left panel) and quantitative intima to media area analysis (right panel) of the carotid arteries subject to balloon-injured and sham-operated 14 days after surgery (n=6, scale bar=100 μm). (**B-C**) Representative immunofluorescence images and quantifications showing the expression of α-SMA, PCNA, OPN and SM22α. Three randomly selected fields for each sample were acquired (n=4, scale bar=100 μm). (**D**) Representative immunofluorescence image showing the maker protein α-SMA expression of the *in vitro* cultured VSMCs (scale bar=100 μm). Three randomly selected fields for each sample were acquired (n=3). (**E**) Representative EdU staining images (left panel, scale bar=50 μm) and quantitative analysis of VSMCs proliferation (right panel) upon PDGF treated or not. Three randomly selected fields for each sample were acquired (n=3). (**F**) Representative scratch images (left panel, scale bar=200 μm) and quantification of VSMCs migration (right panel) upon PDGF treated or not. Three randomly selected fields for each sample were acquired (n=3). (**G**) Representative transwell assay images (left panel, scale bar=100 μm) and quantification of VSMCs migration (right panel) upon PDGF treated or not. Three randomly selected fields for each sample were acquired (n=3). (**H**) RT-qPCR quantification of relative contractile genes, including *α-Sma*, *Sm22α*, *Cnn1* and synthetic genes including *Opn, Myh10* and *Vim* expression upon PDGF treated or not (n = 3). All data were presented as mean ± SD. **p* < 0.05, ***p* < 0.01 and ****p* < 0.001 and ns indicates no significance.


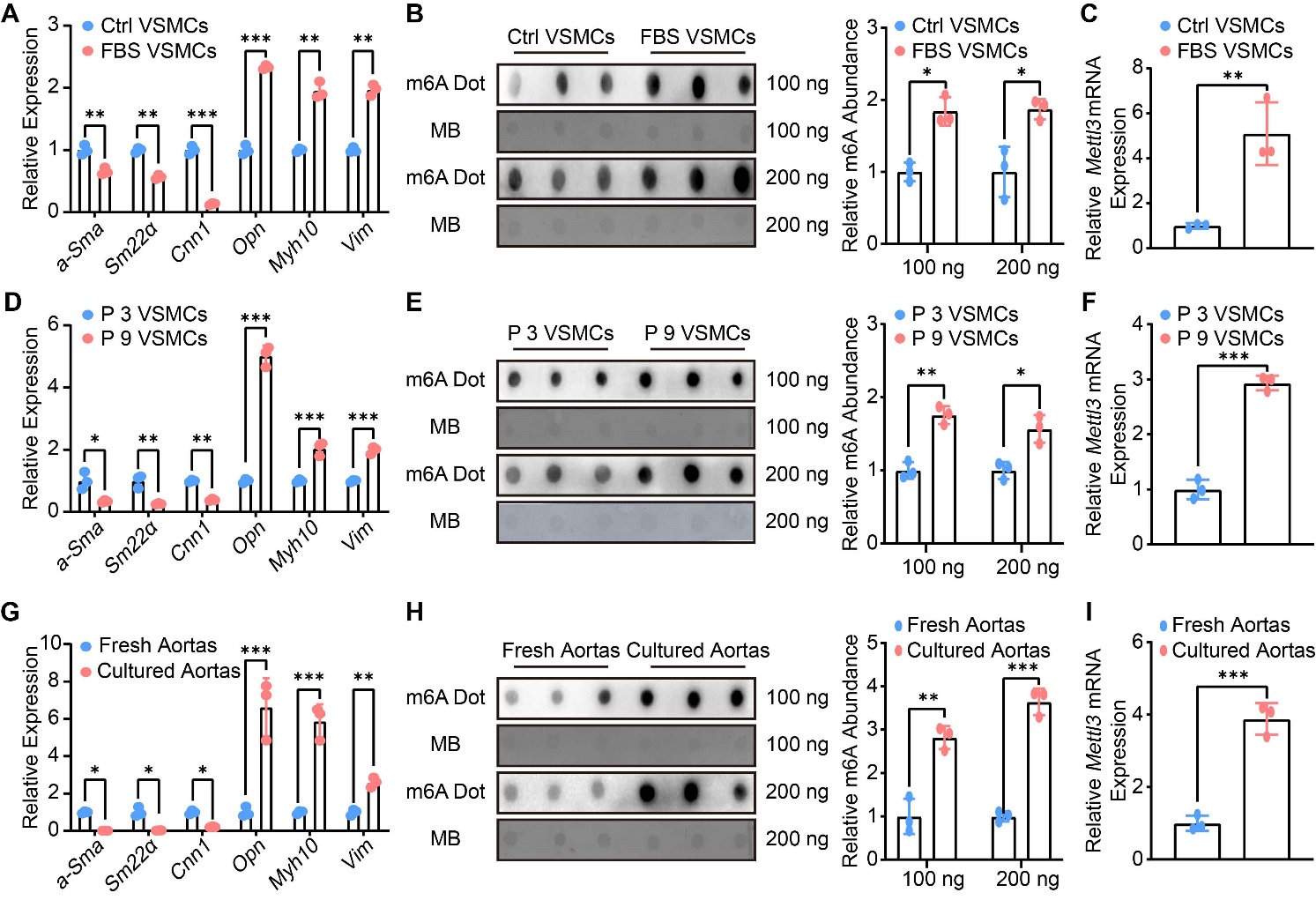


**Supplementary Figure 2.** (**A**) RT-qPCR quantification of relative contractile and synthetic genes expression upon 20% FBS treated or not (n = 3). (**B**) Dot Blot image (left panel) and quantitative analysis of VSMCs m6A (right panel) upon 20% FBS treated or not (n = 3). **(C)** RT-qPCR quantification of relative *Mettl3* mRNA expression upon 20% FBS treated or not (n = 3). (**D**) RT-qPCR quantification of relative contractile and synthetic genes expression in 3^rd^ passage (P 3) and 9^th^ passage (P 9) of VSMCs (n = 3). (**E**) Dot Blot image (left panel) and quantitative analysis of VSMCs m6A (right panel) in P 3 and P 9 VSMCs (n = 3). (**F**) RT-qPCR quantification of relative *Mettl3* mRNA expression in P 3 and P 9 VSMCs (n = 3). (**G**) RT-qPCR quantification of relative contractile and synthetic genes expression in fresh thoracic aortas or cultured thoracic aortas for 3 days (n = 3). (**H**) Dot Blot image (left panel) and quantitative analysis of m6A (right panel) in fresh or cultured thoracic aortas for 3 days (n = 3). (**I**) RT-qPCR quantification of relative *Mettl3* mRNA expression in fresh or cultured thoracic aortas for 3 days (n = 3). All data were presented as mean ± SD. **p* < 0.05, ***p* < 0.01 and ****p* < 0.001.

**
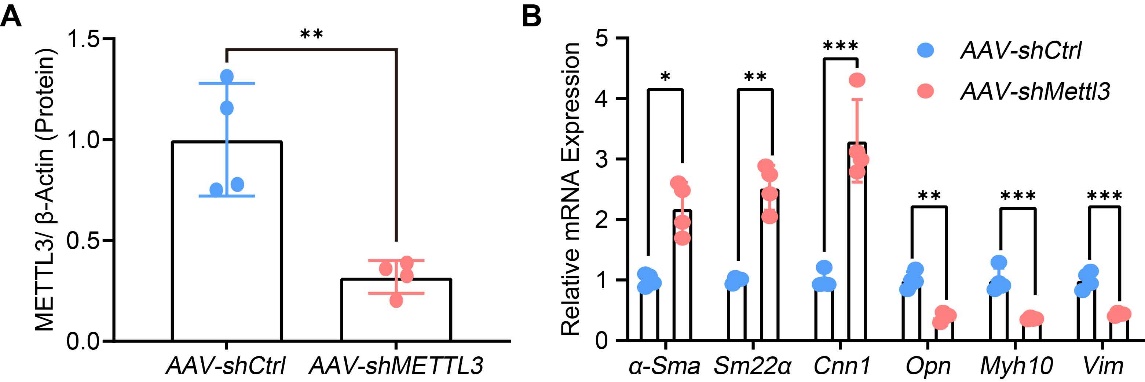
**

**Supplementary Figure 3.** (**A**) Western Blot quantification of carotid artery METTL3 protein expression upon *AAV-shCtrl* or *AAV-shMettl3* treated condition (n = 4). (**B**) Quantitative analysis of VSMCs contractile and synthetic genes expression upon *AAV-shCtrl* or *AAV-shMettl3* treated condition (n = 3). All data were presented as mean ± SD. **p* < 0.05, ***p* < 0.01 and ****p* < 0.001.


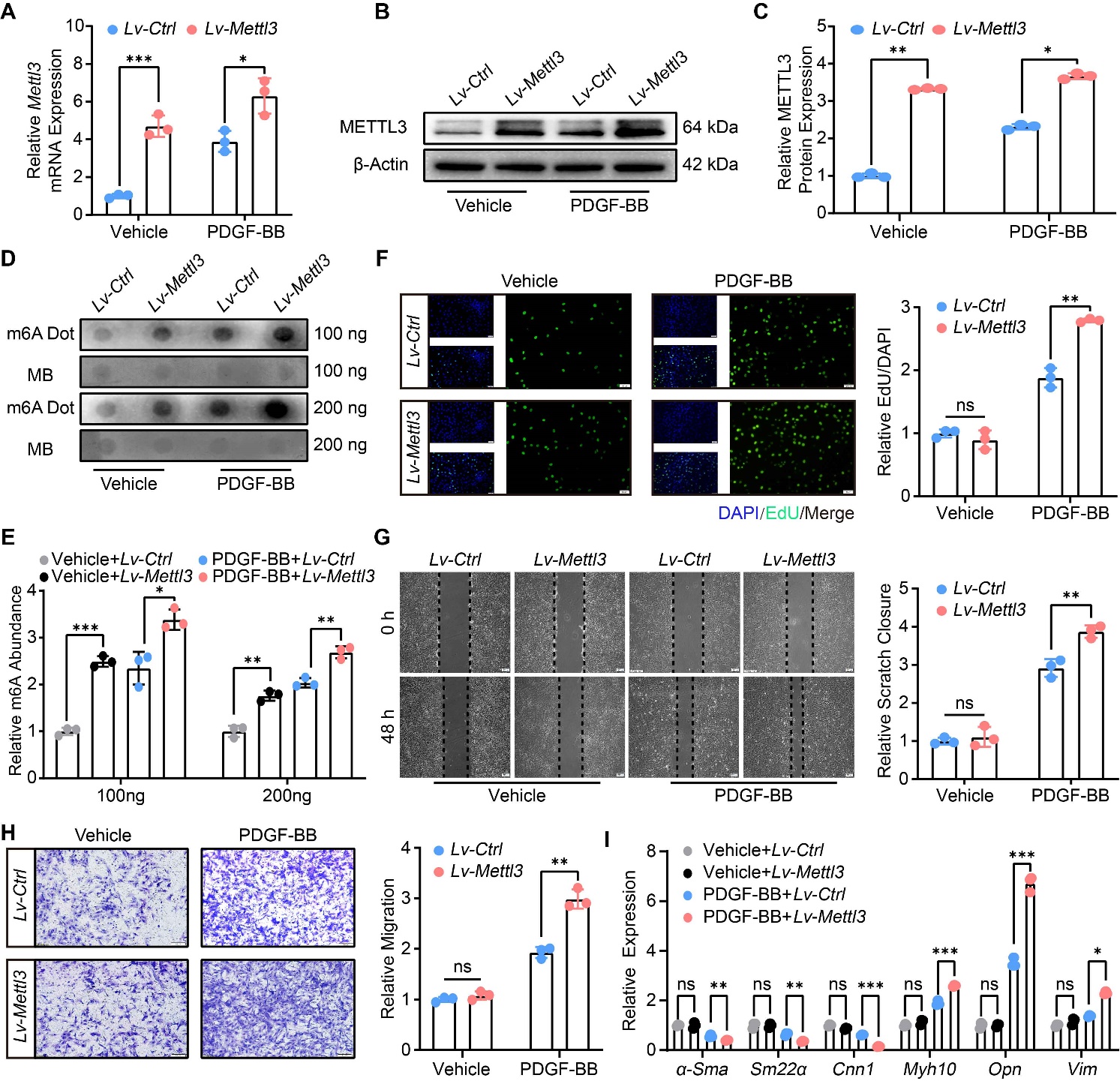


**Supplementary Figure 4.** (**A**) Quantitative analysis of *Mettl3* expression transfected with control lentivirus (*Lv-Ctrl*) and lentivirus target to *Mettl3* (*Lv-Mettl3*) upon PDGF-BB present or not. (**B-C**) Representative Western Blot image and quantification showing METTL3 expression. (**D-E**) Representative Dot Blot image and quantitative analysis of m6A level. (**F**) Representative EdU images (left panel, scale bar=50 μm) and quantification of VSMCs proliferation (right panel). Three randomly selected fields for each sample were acquired. (**G**) Representative scratch images (left panel, scale bar=200 μm) and quantification of VSMCs migration (right panel). Three randomly selected fields for each sample were acquired. (**H**) Representative transwell assay images (left panel, scale bar=200 μm) and quantification of VSMCs migration (right panel). Three randomly selected fields for each sample were acquired. (**I**) Quantitative RT-qPCR analysis of *α-Sma*, *Sm22α*, *Cnn1*, *Opn*, *Myh10* and *Vim* expression. All data were from 3 independent biological replicates and presented as mean ± SD. **p* < 0.05, ***p* < 0.01 and ****p* < 0.001 and ns indicates no significance.
